# Generation and characterization of a zebrafish gain-of-function *ACOX1* Mitchell disease model

**DOI:** 10.3389/fped.2024.1326886

**Published:** 2024-01-31

**Authors:** Quentin Raas, Austin Wood, Tamara J. Stevenson, Shanna Swartwood, Suzanne Liu, Rangaramanujam M. Kannan, Sujatha Kannan, Joshua L. Bonkowsky

**Affiliations:** ^1^ Department of Pediatrics, University of Utah School of Medicine, Salt Lake City, UT, United States; ^2^ Laboratory of Translational Research for Neurological Disorders, Imagine Institute, Université de Paris, INSERM UMR 1163, Paris, France; ^3^ Department of Ophthalmology, Center for Nanomedicine, Wilmer Eye Institute, Johns Hopkins University School of Medicine, Baltimore, MD, United States; ^4^ Department of Anesthesiology and Critical Care Medicine, Johns Hopkins University School of Medicine, Baltimore, MD, United States; ^5^ Center for Personalized Medicine, Primary Children’s Hospital, Salt Lake City, UT, United States

**Keywords:** zebrafish, ACOX1, Mitchell disease, peroxisome, leukodystrophy

## Abstract

**Background:**

Mitchell syndrome is a rare, neurodegenerative disease caused by an ACOX1 gain-of-function mutation (c.710A>G; p.N237S), with fewer than 20 reported cases. Affected patients present with leukodystrophy, seizures, and hearing loss. ACOX1 serves as the rate-limiting enzyme in peroxisomal beta-oxidation of very long-chain fatty acids. The N237S substitution has been shown to stabilize the active ACOX1 dimer, resulting in dysregulated enzymatic activity, increased oxidative stress, and glial damage. Mitchell syndrome lacks a vertebrate model, limiting insights into the pathophysiology of ACOX1-driven white matter damage and neuroinflammatory insults.

**Methods:**

We report a patient presenting with rapidly progressive white matter damage and neurological decline, who was eventually diagnosed with an ACOX1 N237S mutation through whole genome sequencing. We developed a zebrafish model of Mitchell syndrome using transient ubiquitous overexpression of the human ACOX1 N237S variant tagged with GFP. We assayed zebrafish behavior, oligodendrocyte numbers, expression of white matter and inflammatory transcripts, and analysis of peroxisome counts.

**Results:**

The patient experienced progressive leukodystrophy and died 2 years after presentation. The transgenic zebrafish showed a decreased swimming ability, which was restored with the reactive microglia-targeted antioxidant dendrimer-*N*-acetyl-cysteine conjugate. The mutants showed no effect on oligodendrocyte counts but did display activation of the integrated stress response (ISR). Using a novel SKL-targeted mCherry reporter, we found that mutants had reduced density of peroxisomes.

**Conclusions:**

We developed a vertebrate (zebrafish) model of Mitchell syndrome using transient ubiquitous overexpression of the human ACOX1 N237S variant. The transgenic mutants exhibited motor impairment and showed signs of activated ISR, but interestingly, there were no changes in oligodendrocyte counts. However, the mutants exhibited a deficiency in the number of peroxisomes, suggesting a possible shared mechanism with the Zellweger spectrum disorders.

## Introduction

ACOX1 is the rate-limiting enzyme in the first step of fatty acid (FA) beta-oxidation occurring in the peroxisome, a critical pathway for the metabolism/degradation of very long-chain fatty acids (VLCFAs). VLCFAs are primarily synthesized endogenously in the endoplasmic reticulum by elongation and form an essential component of plasma membranes. In the central nervous system (CNS) white matter, the myelin enwrapping axons is a specialized membrane highly enriched in lipids and produced by oligodendrocytes, whose structural and electrical properties are strongly defined by this specific composition. Although constituting a small fraction of total FAs (approximately 5%), VLCFAs are significantly more abundant in myelin lipids (1).

ACOX1 deficiency, previously known as pseudoneonatal adrenoleukodystrophy (ALD), is a rare and severe autosomal recessive disorder characterized by infantile-onset hypotonia, leukodystrophy, seizures, visual and hearing impairment, loss of motor achievements, and progressive gray matter degeneration [resembling Zellweger spectrum disorders (ZSDs)] (2, 3). Leukodystrophies are a heterogeneous group of rare inherited disorders characterized by defects in the production or maintenance of myelin (hypomyelination, dysmyelination, or demyelination). These disorders often cover a broad clinical spectrum, and specific genetic defects can result in early-infantile or late-adult onset. Several leukodystrophies are caused by disrupted VLCFA metabolism, including peroxisomal disorders such as X-linked adrenoleukodystrophy or Zellweger spectrum disorders. In mice, the absence of functional peroxisomes in oligodendrocytes causes progressive subcortical demyelination along with axon degeneration and neuroinflammation (4). Different models of ACOX1 deficiency have brought to light the importance of peroxisome β-oxidation in the regulation of oxidative stress and inflammation in glial and microglial cells (5–7).

The ACOX1 (c.710A>G; p.N237S) gain-of-function mutation (OMIM # 609751.0008) was identified as a putative *de novo* variant causing Mitchell syndrome (OMIM # 618960), an autosomal dominant, progressive degenerative process involving sensorineural hearing loss, polyneuropathy, cognitive decline, and seizures (5). As of May 2023, 20 cases have been reported globally by the Mitchell and Friends Foundation and 4 case reports have been published (8, 9). The N237S substitution has been shown to produce a stabilized active ACOX1 dimer, resulting in dysregulated enzymatic activity, increased oxidative stress, and glial damage (5).

Mitchell syndrome currently lacks a vertebrate model, limiting insights into the role of ACOX1-driven white matter damage and neuroinflammatory insults. Furthermore, the biochemical basis for the ACOX1-dependent oxidative stress damage is unclear. Zebrafish (*Danio rerio*) is an appealing option for a vertebrate Mitchell syndrome model, as zebrafish have been successfully used for modeling peroxisomal disorders and drug screening (10, 11).

We report a case of a patient presenting with rapidly progressive white matter damage, who was eventually diagnosed with an ACOX1 N237S variant through next-generation sequencing and re-analysis. We developed a zebrafish model of Mitchell syndrome with ubiquitous overexpression of the human ACOX1 N237S variant. The transgenic zebrafish showed a decreased swimming ability, which could be restored with dendrimer-*N*-acetyl-cysteine conjugate nanomedicine, and displayed signs of disrupted peroxisome homeostasis.

## Methods

### Ethics approval and consent to participate

The Institutional Review Boards of the University of Utah and Intermountain Healthcare approved this study.

### Zebrafish ethics statement

Zebrafish experiments were performed in strict accordance with guidelines from the University of Utah Institutional Animal Care and Use Committee (IACUC), regulated under federal law (the Animal Welfare Act and Public Health Services Regulation Act) by the U.S. Department of Agriculture (USDA) and the Office of Laboratory Animal Welfare at the NIH, and accredited by the Association for Assessment and Accreditation of Laboratory Care International (AAALAC).

### Sequence analysis

Human and zebrafish ACOX1 amino acid sequences were compared using Clustal Omega and aligned using PRALINE (12, 13).

### Fish stocks and embryo raising

Adult fish were bred according to standard methods. Embryos were raised at 28.5°C in E3 embryo medium and staged by time and morphology. The transgenic fish line used in this paper was Tg(*olig2:dsRed)* (14).

### RT-qPCR

RNA was extracted from groups of 15 larvae at 7 days post-fertilization (dpf) in triplicate using TRIzol reagent according to the manufacturer's instructions (Invitrogen) and PureLink RNA extraction mini kit (Invitrogen). For each sample, 1 µg of total RNA was reverse-transcribed using SuperScript IV Reverse Transcriptase VILO master mix with ezDNase according to the manufacturer's protocol (Invitrogen). Primers for RT-qPCR were designed and efficiency tested according to the MIQE guidelines (15). Primers were as follows: actb forw. 5′-GACAACGGCTCCGGTATG-3′, actb rev. 5′-CATGCCAACCATCACTCC-3′, gapdh forw. 5′-GTGGAGTCTACTGGTGTCTTC-3′, gapdh rev. 5′-GTGCAGGAGGCATTGCTTACA-3′, mbp forw. 5′-AGGGAAAGAGACCCCACCAC-3′, mbp rev. 5′-GAGGAGAGGACACAAAGCTCC-3′, mpz forw. 5′-TACCGTCCAGATGGGGCTAA-3′, mpz rev. 5′-TACCGTCCAGATGGGGCTAA-3′, tnfa forw. 5′-TACCGTCCAGATGGGGCTAA-3′, tnfa rev. 5′-CCCTGGGTCTTATGGAGCGT-3′, gfap forw. 5′-GACTCAATGCTGGCAAAGCC-3′, gfap rev. 5′-CCGCTTCATCCACATCTTGTC -3′ nos2a forw. 5′-AAGTATGCCACCAACGGAGG-3′, nos2a rev. 5′-CGCCGATCACACTACCATCA-3′, cat forw. 5′-TGACAACGTGACCCAAGTGC-3′, cat rev. 5′-GTGTTCTTTTTCCCTTCAGCGT-3′, prdx5 forw. 5′- GATCCCACAGGAGCCTTCAC-3′, prdx5 rev. 5′-TCAGCATGGCATACCTTTGAGA-3′, ddit3 forw. 5′-AAGGAAAGTGCAGGAGCTGA-3′, ddit3 rev. 5′-TCACGCTCTCCACAAGAAGA-3′, atf4 forw. 5′-TTAGCGATTGCTCCGATAGC-3′, atf4 rev. 5′-GCTGCGGTTTTATTCTGCTC-3′, slc7a11 forw. 5′-TGATTGCCATAAGACCCGCAG-3′, slc7a11 rev. 5′-AGTCCAGCTTACGCTCATGC-3′, pparab forw. 5′-CAGGACGAGTTCACCTCCAC-3′, pparab rev. 5′-TCCGACGGAAGAAACCCTTG-3′, pex11a forw. 5′-GGATCGCATTTTCAGGGCAA-3′, pex11a rev. 5′-AAGAGTGCTCTTGGCAGCTT-3′, pex11b forw. 5′-GAGCCTCACCAGAAAATTGATGT-3′, pex11b rev. 5′-TTTTTCCGGCCCACAGTACA-3′, pex11g forw. 5′-CTAAATCTGACCGCTGGTGGG-3′, pex11g rev. 5′-AAAACACTTCCTCCCTGCTCT-3′, nbr1b forw. 5′-AACCAAATCAGCCTGACTCC-3′, and nbr1b rev. 5′-CTGCATCCTCTGGGAGCTTT-3′.

All data were normalized to both *actb* and *gapdh* expression levels using the ΔΔCt method in accordance with the MIQE guidelines (15).

### Establishment of the Tg (β-actin:mCherry-SKL) line

The peroxisomal targeting signal 1 (PTS1) tripeptide signal SKL was added through amplification of the mCherry CDS using the following primers: 5′-GGGGACAAGTTTGTACAAAAAAGCAGGCTGCCGCCACCATGGTGAGCAAGGGCGAGGA-3′ and 5′-GGGGACCACTTTGTACAAGAAAGCTGGGTATTAGAGCTTGCTCTTGTACAGCTCGTCCATGC-3′. The mCherry-SKL CDS was cloned into pDONR221, and expression clones were built using the Tol2 kit and recombination reactions with Gateway plasmids.

### Human ACOX1 N237s targeted mutagenesis and construct generation

The full-length coding DNA sequence encoding for human ACOX1b (NM_007292) was amplified using primers 5′-ATGAACCCGGACCTGCGCAG-3′ and 5′-TCAGAGCTTGGACTGCAGTG-3′ and cloned into pDONR221. pME-ACOX1^N237S^ was generated by targeted mutagenesis using the pME-ACOX1 WT sequence and primers 5′-GAGATAGACAGTGGCTACCTC-3′ and 5′-ATCATAACCAAATTTGGGG-3′.

Expression clones were built using the Tol2 kit and recombination reactions with Gateway plasmids (Thermo Fisher) (16). The identity of the constructs was confirmed by restriction enzyme digests, and the ACOX1 and ACOX1N237S coding sequences were confirmed by sequencing both strands. Specific plasmids used for cloning were p5E-β-actin, pME-hACOX1, pME-hACOX1N237S, and p3E-IRES-EGFP, all integrated into pDestTol2pA2. Embryos were microinjected at the one-cell stage with 50 pg of plasmid DNA and 50 pg of Tol2 transposase mRNA.

### Behavior analysis

Larval behavior analysis was performed on 7 dpf larvae in 96-well square-bottom plates (Krackeler Scientific) using video analysis software (Noldus EthoVision). For spontaneous behavior, animals were transferred at 6 dpf to a 96-well plate and kept at 28.5°C overnight. At 7 dpf, the plate was placed on the video imaging system, animals were allowed to adapt in the dark for 10 min, and then recording was performed for 5 min (1 min dark and 4 min light).

### Microscopy and image analysis

Transgenic and/or injected zebrafish larvae were treated with phenylthiourea from 24 hpf and were anesthetized with tricaine (MS222) at age 5–7 dpf before live mounting laterally in E3 embryo water containing 2% low melting agar on a glass slide with a #0 coverslip. For live imaging of neural masts, 5-dpf Tol2-β-Actin:mCherry-SKL microinjected larvae were treated with DAPI 1:1,000 in E3 embryo water for 1 h. For each larva, three neural masts corresponding to groups of 5–10 cells, lively stained with DAPI and evenly distributed between head and tail, were imaged. Z-stacks were acquired using a Zeiss LSM700 confocal microscope with x20 or oil x63 objectives and adequate z steps (0.4 µm on x63). Confocal stacks were projected in ImageJ, and images were composed with Adobe Photoshop and Illustrator.

Z-stacks were opened in Imaris and reconstructed into a multi-channel 3D model. The surface creation tool was used to generate an ROI. In the spot detection wizard, the mCherry/568 channel was used as the source channel. The size and shape of the generated surface were a direct map of the intensity distribution of the peroxisomal mCherry signal as detected by Imaris. The diameter for spot detection was set at 0.3 µm, and the same intensity threshold was kept for spot detection of all images. For each z-stack representing one neural mast, measured average variables, including the average distance to the nine nearest neighbors, were used for quantification.

### Statistical analysis

The normality of all data was assessed using the Shapiro–Wilk test of normality, and the equality of variances was assessed using Levene's test. Statistical significance was set at *p* < 0.05. Statistical analyses for zebrafish experiments were performed using Prism8 software (GraphPad). Standard deviation was represented by error bars on dot plots or bar graphs. Student's *t*-test was used for two-way comparisons; comparisons between three or more groups were performed with ANOVA with *post-hoc* Tukey's HSD between individual means.

## Results

### Case report

**Figure 1:**
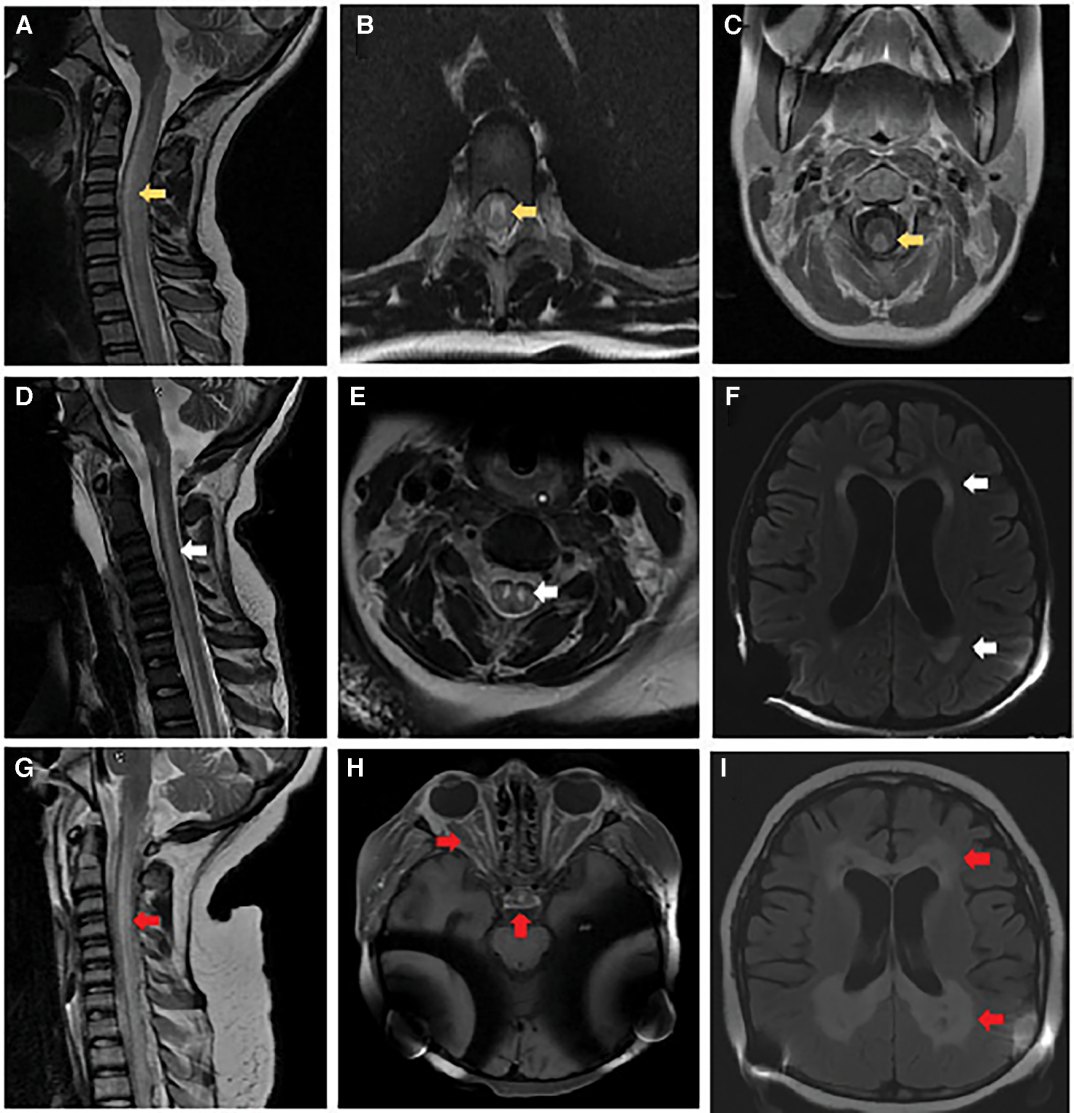
Brain and spinal cord imaging. At initial presentation: (**A**) MRI cervical spine sagittal T2-weighted image with hyperintense signal involving the central gray matter, dorsal and lateral tracts of the cervical and thoracic spinal cord (yellow arrow). (**B**) MRI thoracic spine axial T2-weighted image with hyperintense signal involving primarily gray matter (yellow arrow). (**C**) MRI cervical spine axial post-gadolinium T1-weighted image with contrast enhancement of the dorsal white matter tracts (yellow arrow). Follow-up at 6 months: (**D**) MRI cervical spine sagittal T2-weighted image with re-demonstration of central gray matter hyperintensity of the cervical and thoracic spinal cord with associated volume loss (white arrow). (**E**) MRI cervical spine axial T2-weighted image with increased hyperintense signal involving primarily gray matter and volume loss within the gray matter tracts (white arrow). (**F**) MRI brain axial T2-weighted FLAIR image with diffuse parenchymal volume loss and nearly symmetric bilateral periventricular and deep white matter signal abnormality in the frontal, parietal, and occipital lobes (white arrows). Obtained 2 weeks before death: (**G**) MRI cervical spine sagittal T2-weighted image with recurrent non-enhancing hyperintense signal involving primarily the dorsal central aspect of the spinal (red arrow). (**H**) MRI brain axial T1-weighted imaging post-gadolinium with contrast enhancement involving the optic chiasm, prechiasmatic optic nerves, and bilateral optic tracts within the suprasellar region (red arrows). (**I**) MRI brain axial T2-weighted FLAIR image with progressive bilateral periventricular white matter signal abnormality with central areas of white matter necrosis (red arrows).

An 11-year-old girl with a 2-year history of progressive bilateral sensorineural hearing loss of unknown etiology presented to the emergency department at Primary Children's Hospital in Salt Lake City, Utah, with 4 weeks of difficulty walking, resulting in multiple falls. She had a fever and strep throat 5 weeks prior to the presentation. On initial presentation, her neurologic examination was remarkable for 4/5 strength and decreased sensation to pinprick throughout the lower extremities, brisk lower extremity reflexes with bilateral ankle clonus, and a wide based gait. She was diagnosed with longitudinally extensive transverse myelitis (LETM) based on magnetic resonance imaging (MRI) findings (Figures 1A–C) and was started on empiric treatment with intravenous high-dose steroids and intravenous immunoglobulin (IVIg). Her clinical course was complicated by seizures secondary to posterior reversible encephalopathy syndrome, along with a spontaneous bowel perforation and pseudomonal urinary tract infection (UTI). Over the next 4 months, the patient faced multiple hospital admissions, with an increasing burden of demyelinating lesions (Figures 1D–F) and a waxing and waning ichthyotic rash. Pathology results from skin biopsy were non-diagnostic. A broad diagnostic evaluation, including repeated lumbar punctures, revealed only mildly elevated CSF protein (66 mg/dl, reference range 15–60 mg/dl). Extensive autoimmune screening with complements, antistreptolysin O (ASO) titers, DNAse antibody, aquaporin-4 (AQP4), myelin oligodendrocyte glycoprotein (MOG), celiac panel, aldolase, Epstein–Barr virus (EBV) antibody panel, and cytomegalovirus (CMV) yielded unremarkable results. Genetic testing, including whole genome sequencing (WGS), did not find a unifying diagnosis for her array of symptoms. Subsequently, she developed alopecia and permanent vision loss with ongoing waxing and waning ichthyotic rash. Based on updated neuroimaging findings and clinical symptoms, the presumptive diagnosis shifted from LETM to antibody-negative neuromyelitis optica spectrum disorder (NMOSD), and she was initiated on treatment with rituximab, azathioprine, and steroids. She experienced mild improvement in symptoms and returned home with stable disease symptoms for 9 months.

She was then re-admitted to the hospital with urosepsis, and new demyelinating lesions were found (Figures 1G–I); persistently elevated ferritin, C-reactive protein (CRP), and interleukin-2 (IL-2) receptor levels raised concerns of hemophagocytic lymphohistiocytosis (HLH) with CNS involvement, as well as possible macrophage activation syndrome (MAS), consequently, treatment with anakinra and cyclosporine was initiated. Considering her rapid deterioration and the absence of a unifying diagnosis, her previous WGS was re-analyzed and found to have a *de novo* heterozygous pathogenic *ACOX1* (p.N237S) gain-of-function mutation. Enteral treatment with N-acetyl-cysteine (NAC) was started at 1 g every 6 h. Unfortunately, the patient did not exhibit any clinical improvement after starting NAC therapy and ultimately succumbed to the condition.

### Zebrafish model of Mitchell syndrome

**Figure 2:**
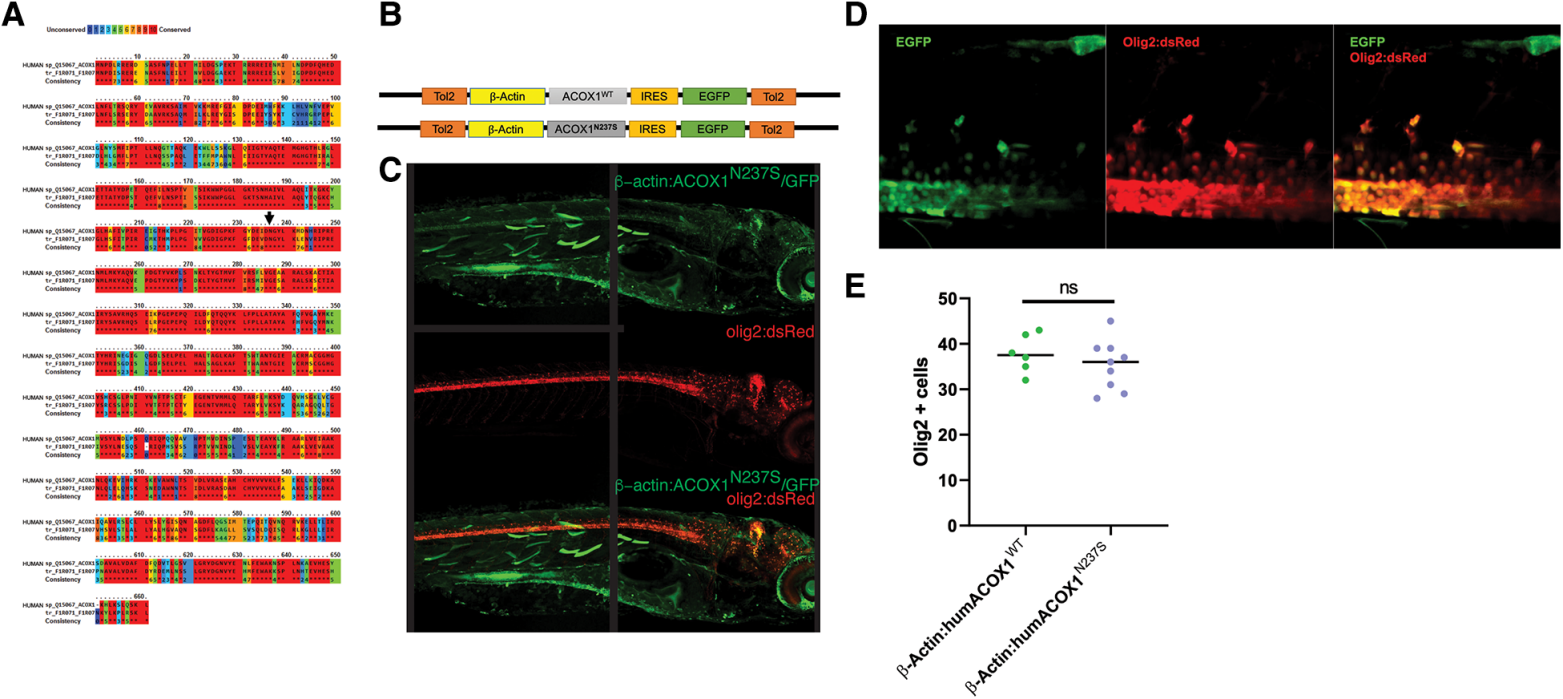
(**A**) Zebrafish Acox1 protein is highly conserved compared to human ACOX1. The degree of amino acid similarity is indicated by color, with red being identical and blue being non-conserved, as visualized using PRALINE. (**B**) Schematic diagram of expression constructs used in zebrafish experiments. (**C**) Confocal z-stack images of the expression of the construct in live zebrafish larvae; immunohistochemistry for anti-GFP, and anti-RFP in the Tg(*olig2:dsRed*) line. Dorsal to top, rostral to the right; age 3 dpf. Note: the black line in the middle of the figure indicates where the image was stitched together. (**D**) Confocal z-stack images, spinal cord, rostral to the right, dorsal to the top. Expression of N237S is indicated by tagged GFP, and mature oligodendrocytes are labeled by olig2:dsRed. (**E**) Quantification shows no effect on oligodendrocyte numbers.

The single *acox1* orthologue in zebrafish (*Danio rerio*) has 70% amino acid sequence identity to human *ACOX1* (Figure 2A). We observed the conservation of the enzyme binding and active sites and the asparagine 237 residue in the zebrafish ortholog, suggesting the conservation of its biochemical role in zebrafish. We amplified and cloned the cDNA encoding the human *ACOX1b* isoform, generated a mutant N237S by directed mutagenesis, and then confirmed the correct sequence by Sanger sequencing. We then used the Tol2 transposon system to enable the insertion of the mutant or WT ACOX1 expression clone. Ubiquitous overexpression of the human mutant or WT ACOX1 was obtained using a β-actin promoter (Figure 2B). To avoid disruption of the C-terminal peroxisomal targeting signal, the sequence was terminated by a stop codon and no tag was added, but expression of an EGFP fluorescent reporter was enabled using an internal ribosome entry site (IRES). While the use of bi-cistronic reporters such as IRES element sometimes results in poor expression in zebrafish (16), embryos injected at the one-cell stage show heterogeneous expression of the GFP reporter in various tissues, including CNS olig2+ cells, at 5 dpf (Figure 2C). The survival to adulthood of the injected zebrafish was unaffected.

Although expression of the transgene was observed in olig2-expressing oligodendrocyte precursor cells (OPCs), the cell number was not significantly affected in ACOX1 N237S-expressing zebrafish compared to their ACOX1 WT-expressing controls (Figures 2D,E).

**Figure 3:**
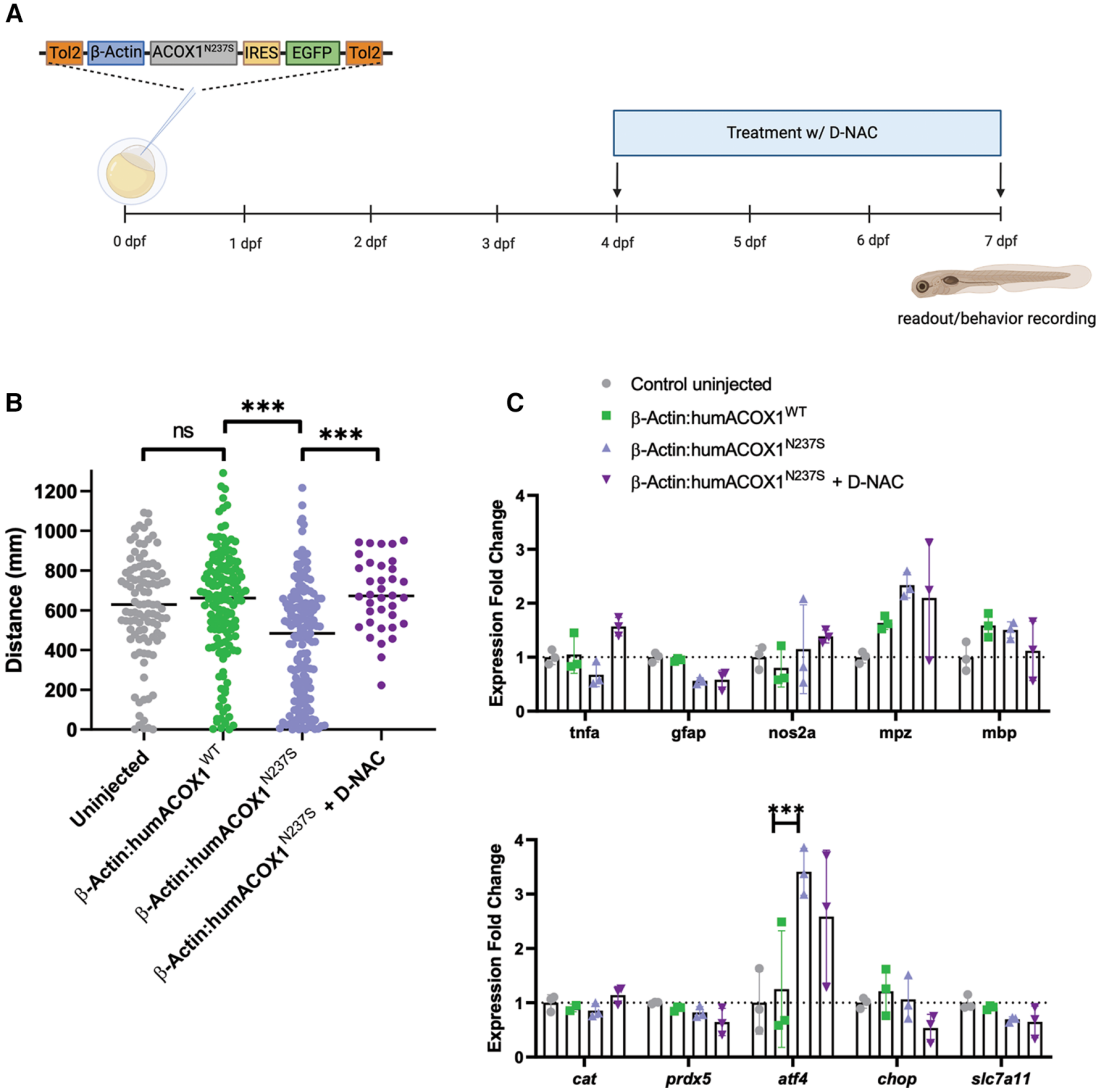
Characterization of zebrafish mutant experiments by overexpression of human ACOX1 N237S. (**A**) Schematic diagram of experiments. (**B**) Zebrafish show abnormal motor (swimming) behavior, which is rescued by D-NAC. ***p < 0.001. (**C**) qRT-PCR characterization of transcripts. Expression of mutant form does not impact myelin transcripts or general inflammatory markers but does activate the ISR (*atf4*).

Considering the prominent role of oxidative stress in Mitchell disease, we investigated the effect of an antioxidant using a dendrimer conjugated N-acetyl-cysteine (D-NAC), a technique that showed improved CNS penetration and promising results in other models of leukodystrophies (17). The motor behavior of injected larvae was recorded using an automated tracking system (Figure 3A). The swimming distance and velocity of ACOX1 N237S zebrafish larvae were found to be decreased compared to control larvae overexpressing the WT ACOX1 at 7 dpf (Figure 3B). Larvae incubated for 48 h (from 5 to 7 dpf) with D-NAC in the embryo water showed a restored motor phenotype at 7 dpf.

Next, we examined whether the expression of mutant ACOX1 N237S in zebrafish could affect the inflammatory response in the larvae CNS. We measured the expression of key pro-inflammatory genes, such as *tnfa*, *gfap*, or the gene encoding the zebrafish inducible NO synthase *nos2a*, by RT-qPCR at 7 dpf. No significant change in expression was observed for any of these genes upon expression of the mutant ACOX1 compared to their control. Similarly, the transcript levels of zebrafish major myelin proteins, *mpz* and *mbp*, were not significantly different, and expression of the zebrafish antioxidant enzymes catalase (*cat*) and *prdx5* was also unaffected (Figure 3C). Interestingly, we observed an increased expression of the integrated stress response marker *atf4* in zebrafish larvae overexpressing the mutant ACOX1 at this stage. However, the expression of ATF4 downstream effectors *ddit3* (CHOP) or *slc7a11*, involved in the regulation of cell death or oxidative stress, respectively, was unaffected. Moreover, the increased expression of *atf4* was not corrected after treatment with D-NAC in mutant ACOX1-expressing larvae (Figure 3C).

**Figure 4:**
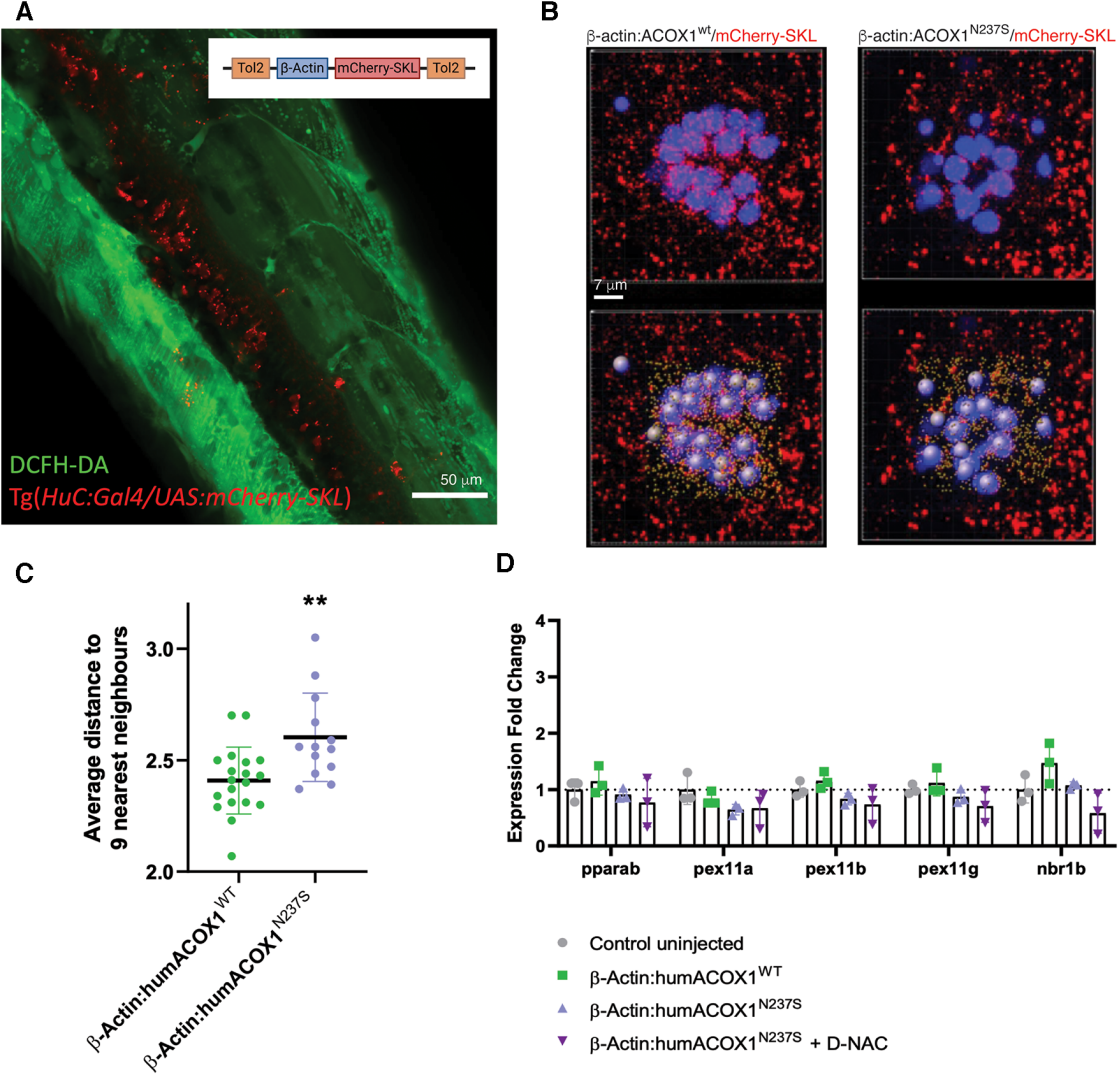
Zebrafish peroxisomal reporter construct and characterization of effects on peroxisomes. (**A**) Schematic of the mCherry-SKL construct and transient expression in zebrafish. Confocal z-stack, lateral view of the trunk, live 3 dpf larva, green channel, DCFH-DA (dacetyldichlorofluorescein). (**B**) Live confocal images and paired IMARIS (3D reconstruction) image (below), of mCherry-SKL (red) containing peroxisomes in the hair cells (blue) of larval zebrafish neuromast at 6 dpf in zebrafish overexpressing WT or N237S ACOX1. (**C**) Size and distance to nearest neighbors in zebrafish overexpressing WT or N237S ACOX1, showing a decreased density of peroxisomes in these cells. (**D**) qRT-PCR for expression of genes that affect the abundance of peroxisome: *pparab*, *pex11a*, *pex11b*, *pex11g*, and *nrb1b*.

Peroxisome homeostasis is a dynamic process, and oxidative stress has previously been reported to activate peroxisome autophagy (pexophagy) (18). Peroxisome features in the ACOX1 N237S zebrafish were investigated using a novel transgenic model expressing a mCherry tagged with the tripeptide PTS1-SKL (Figure 4A). We performed live imaging following transient injections of mCherry-SKL containing peroxisomes in the hair cells of larval zebrafish neuromast at 6 dpf. We used 3D reconstruction to measure specific properties of peroxisomes, including their size and distance to nearest neighbors, in zebrafish overexpressing the WT or N237S ACOX1 constructs (Figure 4B). This analysis revealed a decreased density of peroxisomes in these cells, suggesting decreased biogenesis or increased peroxisome autophagy (Figure 4C).

We finally measured the expression of genes that have been shown to affect the abundance of peroxisomes, including genes controlling fission and proliferation of peroxisomes, pparab, pex11a, pex11b, pex11g, or the autophagy cargo receptor nrb1b. We failed to identify any significant change in the expression of molecular effectors, possibly explaining the difference in peroxisome abundance (Figure 4D).

## Discussion

We report a new case of Mitchell syndrome and its clinical course including MRI findings. Using transient global overexpression of the N237S human ACOX1 cDNA in zebrafish, we are able to recapitulate key disease features. Furthermore, we show that major effects of the gain-of-function ACOX1 include activation of the ISR and reduction in the number of peroxisomes.

ACOX1 is the rate-limiting enzyme in the first step of fatty acid beta-oxidation occurring in the peroxisome, a critical pathway for the metabolism/degradation of VLCFAs. The shortening of VLCFAs requires a dehydrogenation, catalyzed by ACOX1, that uses FAD to convert Acyl-CoA into 2-trans-enoyl-CoA. The FADH2 produced is then reoxidized with molecular oxygen, which results in the production of hydrogen peroxide. Peroxisomes, which take their name from their capacity to produce and degrade hydrogen peroxide, are the major site harboring catalase activity in animals and play a key role in the metabolism of harmful oxidative species and by-products. Three acyl-CoA oxidases have been characterized and show distinct substrate specificities within the peroxisome. ACOX1 is responsible for the oxidation of straight-chain fatty acids, while ACOX2 is involved in bile acid biosynthesis, and both ACOX2 and ACOX3 are involved in the degradation of the branched-chain fatty acids (19). Due to alternative splicing, the human *ACOX1* gene produces two isoforms, ACOX1a and ACOX1b, of the same length, with relatively similar activity and tissue distribution (20). While the substrate specificity of each isoform remains unclear, ACOX1b would possibly show a higher activity toward VLCFA (21).

The accumulation of VLCFA represents a major biochemical marker of peroxisomal disorders, including X-linked adrenoleukodystrophy, Zellweger spectrum disorders, and ACOX1 deficiency. Interestingly, patients presenting with the ACOX1 (p.N237S) gain-of-function mutation have healthy VLCFA levels, suggesting a distinct pathological mechanism that does not revolve around abnormal lipid accumulation. Interestingly, patients with Mitchell syndrome may present with a skin rash or ichthyosis similar to that observed in other conditions with impaired VLCFA production, such as *ELOVL1* deficiency. However, in contrast to *ELOVL1* deficiency, skin biopsies showed no evidence of lipid inclusions (9, 22).

An interesting aspect of our work is that we did not see any direct effect on oligodendrocyte numbers. This is in contrast to the observation of a loss of rat Schwann cells induced by overexpression of the N237S variant (5). One possibility is that the CNS oligodendrocytes and PNS Schwann cells show differential susceptibility to the variant.

Alternatively, we favor that the leukodystrophy and other aspects of Mitchell disease progression may reflect a “2 hit” model, more similar to the cerebral inflammatory disease observed in some X-linked ALD patients (23). Only some ALD patients develop the cerebral demyelinating subtype despite all having the same biochemical signature (elevated VLCFAs). Furthermore, patients with the same variant genotype can have different cerebral phenotypes. Based on reports and pathophysiological characteristics of the disease and of pathology (23–25), the risk for cerebral ALD appears to be partly from an underlying genetically determined risk and then a secondary second effect. Similarly, for most Mitchell patients, disease onset is after many asymptomatic years of life (5 years in this case) (26). Furthermore, in Mitchell syndrome, all reported patients have the same genetic variant but different ages of onset and different rates of progression of CNS leukodystrophy.

Our findings of a reduction in the number of peroxisomes and activation of the ISR in Mitchell syndrome highlight shared characteristics with the ZSDs. ZSDs are caused by mutations in the *PEX* genes that interfere with peroxisome biogenesis or function; ZSD patients exhibit impaired function or absent or reduced numbers of peroxisomes (27, 28). It will be important to test whether the observation of peroxisome deficiency in the zebrafish model is recapitulated. Furthermore, the reasons for the deficiency in the number of peroxisomes in Mitchell syndrome are unclear. For example, excess H_2_O_2_ production in peroxisomes, a feature of Mitchell syndrome, is alone not sufficient to cause peroxisome loss (29). Another shared feature is that both Mitchell syndrome and ZSDs have ISR activation (30).

The proliferation of peroxisomes in mice cochlear hair cells was identified as a protective mechanism in response to oxidative stress involving the peroxisomal protein Pejvakin. Pejvakin-deficient mice display impaired antioxidant activity of peroxisomes and hypervulnerability to sound exposure (31). Here, we noticed a decreased peroxisome density in zebrafish neuromast hair cells that could reflect an abnormal response to oxidative stress associated with the *ACOX1* gain-of-function mutation. Whether such mechanisms could play a role in the hearing loss frequently observed in Mitchell disease patients remains to be established.

Currently, there are no treatments for Mitchell syndrome. The patient we report on was trialed on immunomodulators and immunosuppressives, but disease progression continued unabated. Recent studies have underscored the potential of reactive microglia/macrophage targeting enabled by hydroxyl PAMAM dendrimers, with promising animal and human studies on the D-NAC conjugate used in this study (17, 32, 33). This dendrimer-drug nanomedicine showed promise in targeting microglia in mouse models of leukodystrophy and zebrafish models of neurodegeneration (34). A recently completed phase 2a trial showed that D-NAC (OP-101) is safe in humans, revealing survival benefits and that it significantly attenuated the brain injury biomarkers associated with severe COVID-19 (35). Although NAC was tried in the patient described above, it is known that NAC has poor bioavailability and poor brain penetration, which may have made it ineffective (ref). Our studies have previously shown that cysteine is transported into cells by the xCT transporter (cystine-glutamate antiporter), which can increase local excitotoxicity (36). D-NAC bypasses this antiporter and is taken up specifically into microglia/macrophages involved in inflammation and oxidative stress, thereby avoiding the associated side effects and increasing the cellular concentration of the drug at the site of inflammation. Our finding of the utility of D-NAC in the zebrafish model suggests that specifically targeting neuroinflammation and oxidative stress in Mitchell syndrome may have potential as a novel therapy.

In summary, this study expands our understanding of the clinical phenotype of Mitchell syndrome and the lack of efficacy of immunosuppressives. The zebrafish model offers a promising approach to more thoroughly explore disease pathology and to test the efficacy of potential therapies.

## Data Availability

The original contributions presented in the study are included in the article/Supplementary Material, further inquiries can be directed to the corresponding author.
